# Recognizing and managing sepsis: what needs to be done?

**DOI:** 10.1186/s12916-015-0335-2

**Published:** 2015-04-27

**Authors:** Donald M Yealy, David T Huang, Anthony Delaney, Marian Knight, Adrienne G Randolph, Ron Daniels, Tim Nutbeam

**Affiliations:** University of Pittsburgh, Pittsburgh, PA USA; UPMC, 200 Lothrop St., Pittsburgh, PA 15213-2582 USA; Malcolm Fisher Department of Intensive Care Medicine, Level 6, Acute Services Building, Royal North Shore Hospital, Reserve Road, St. Leonards, Sydney, NSW 2065 Australia; Northern Clinical School, Sydney Medical School, University of Sydney, Reserve Road, St. Leonards, Sydney, NSW 2065 Australia; Department of Epidemiology and Preventative Medicine, Australian and New Zealand Intensive Care Research Centre, Monash University, The Alfred Centre, 99 Commercial Road, Melbourne, VIC 304 Australia; National Perinatal Epidemiology Unit, Nuffield Department of Population Health, University of Oxford, Old Rd Campus, Oxford, OX3 7LF UK; Department of Anesthesia, Perioperative and Pain Medicine, Critical Care Division, Boston Children’s Hospital, Bader 634, 300 Longwood Avenue, Boston, MA 02115 USA; Department of Anesthesia, Harvard Medical School, Boston, MA 02115 USA; Heart of England NHS Foundation Trust, UK Sepsis Trust, Sutton Coldfield, West Midlands, B72 1NE UK; Department of Emergency Medicine, Derriford Hospital, Plymouth, DL6 8DH UK; University of Plymouth, Drake Circus, Plymouth, Devon PL4 8AA UK

**Keywords:** Antibiotics, Emergency medical services, Pediatrics, Pregnancy, Resuscitation, Sepsis

## Abstract

Sepsis is associated with significant morbidity and mortality if not promptly recognized and treated. Since the development of early goal-directed therapy, mortality rates have decreased, but sepsis remains a major cause of death in patients arriving at the emergency department or staying in hospital. In this forum article, we asked clinicians and researchers with expertise in sepsis care to discuss the importance of rapid detection and treatment of the condition, as well as special considerations in different patient groups.

## The key role of early care in sepsis

**Figure 1:**
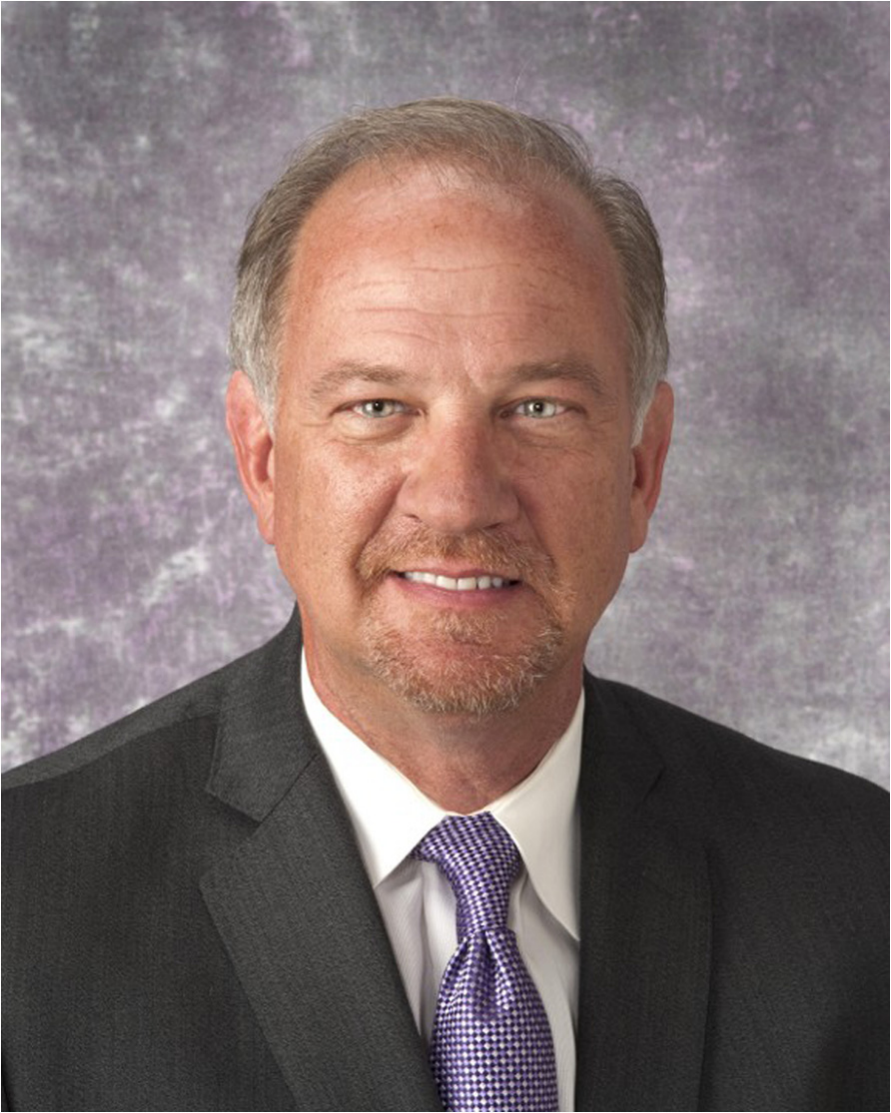
**Donald M. Yealy, MD, is Professor and Chair of Emergency Medicine at the University of Pittsburgh and University of Pittsburgh Medical Center.** He has focused on clinical decision-making and the early care of many life-threatening conditions, including community-acquired pneumonia, sepsis, acute heart failure, and respiratory failure. He has over 300 scientific publications and was awarded the highest recognitions from the American College of Emergency Physicians and the Society for Academic Emergency Medicine for his research, teaching, and leadership.

**Figure 2:**
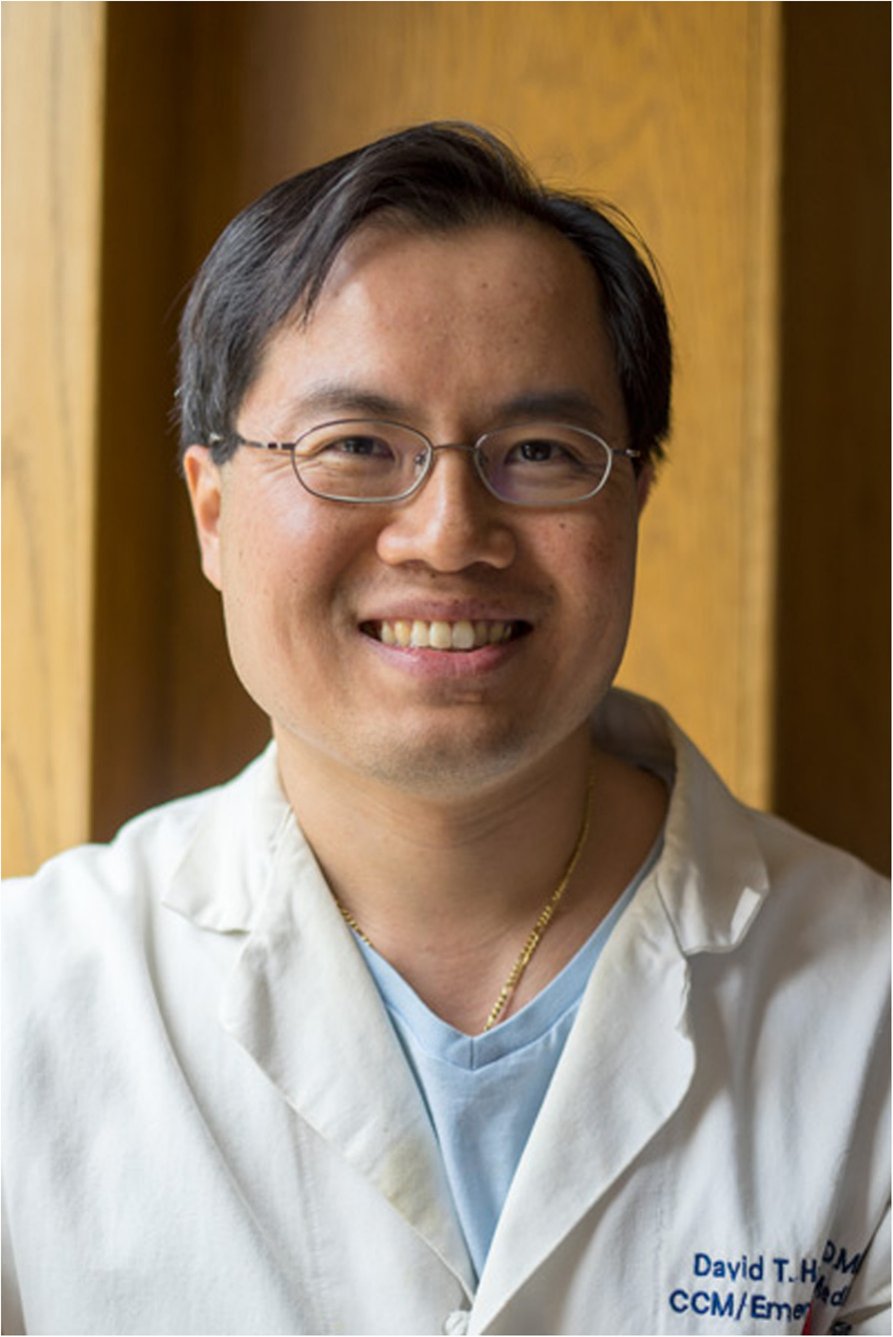
**David T. Huang, MD, MPH, has focused on biomarkers and resuscitation of infection and sepsis, early treatment of acute respiratory distress syndrome, and emergency medicine-critical care medicine physician demographics and education.** He is a Fellow of the American College of Emergency Physicians and the American College of Critical Care Medicine, and Director of MACRO (Multidisciplinary Acute Care Research Organization) at the University of Pittsburgh.

Donald M. Yealy (Figure 1) and David T. Huang (Figure 2)

**Figure 3:**
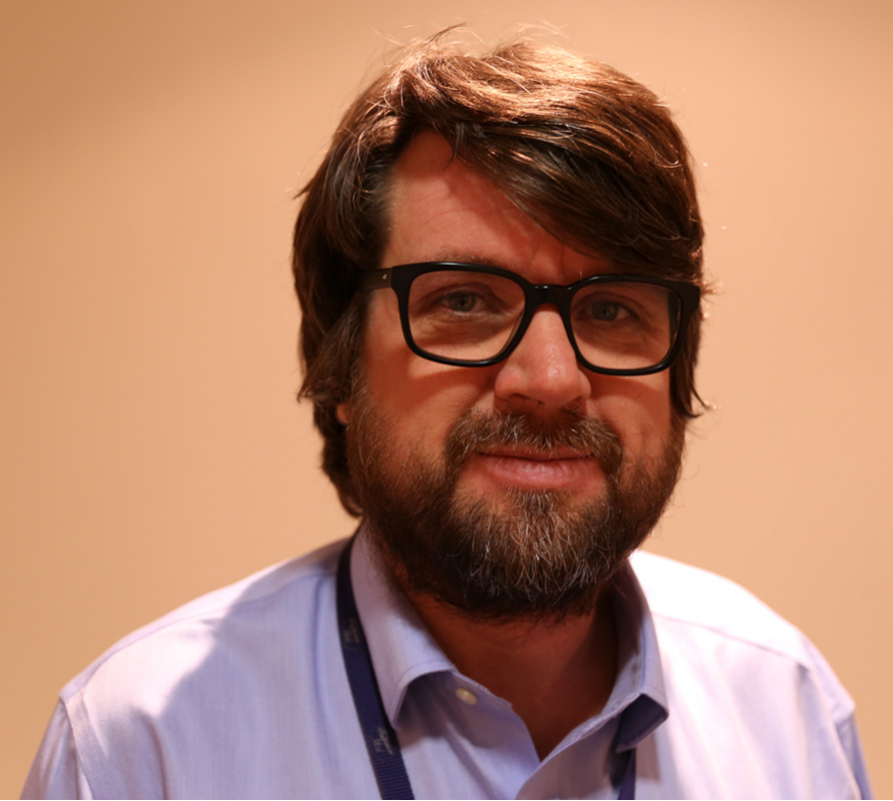
**Anthony Delaney, MBBS, MSc, PhD, FACEM, FCICM, is a Fellow of the Australasian College for Emergency Medicine and the College of Intensive Care Medicine.** He is a Senior Staff Specialist in the Malcolm Fisher Department of Intensive Care Medicine at Royal North Shore Hospital, a Senior Lecturer at Sydney Medical School, University of Sydney, and an adjunct Senior Research Fellow at the Australian and New Zealand Intensive Care Research Centre, in the Department of Epidemiology and Preventative Health at Monash University.

**Figure 4:**
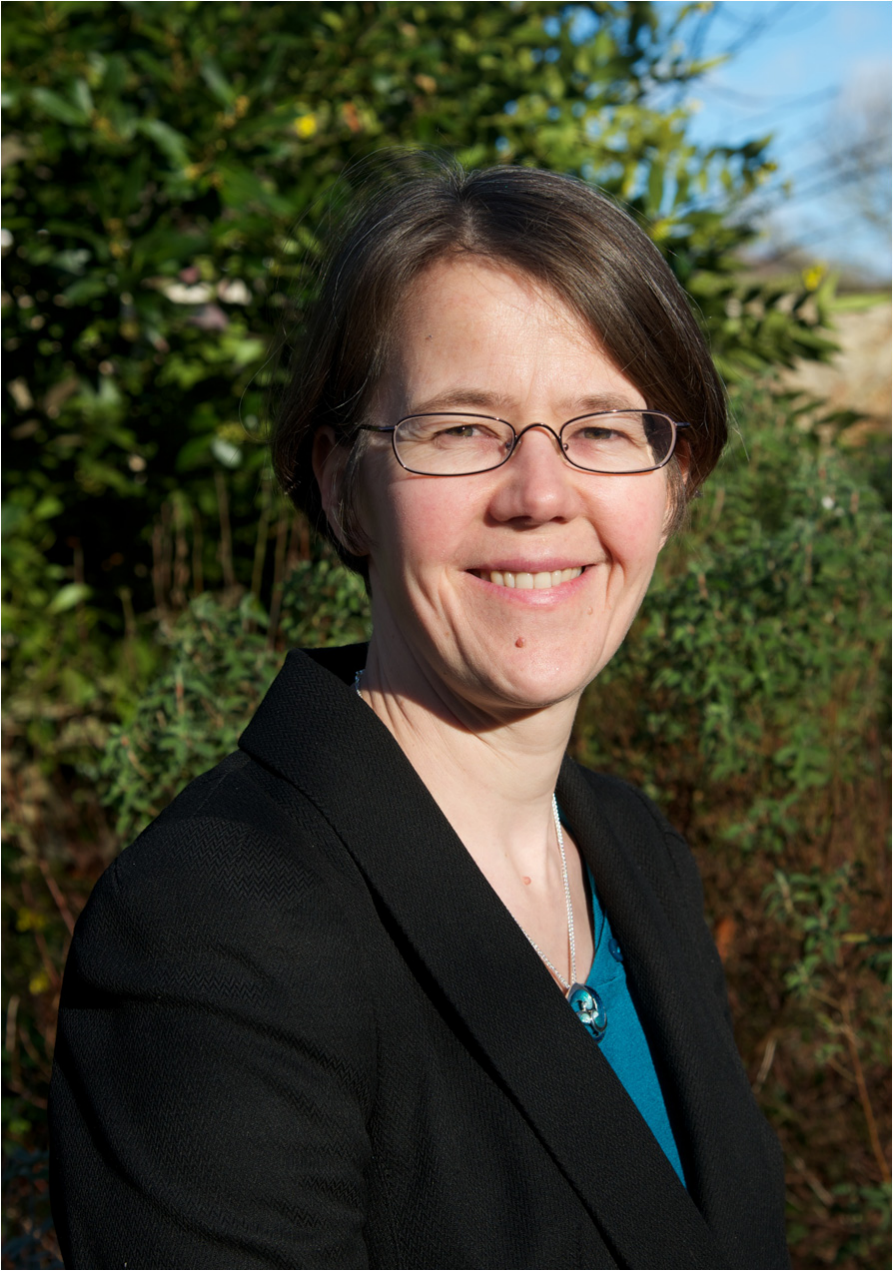
**Marian Knight is Professor of Maternal and Child Population Health, NIHR Research Professor and Honorary Consultant in Public Health at the National Perinatal Epidemiology Unit, University of Oxford.** Her research interests include severe maternal morbidity and the use of population-based observational studies to address key clinical questions in obstetrics. Since 2012, she has led the UK Confidential Enquiries into Maternal Deaths and Morbidity, which most recently focused on maternal sepsis.

**Figure 5:**
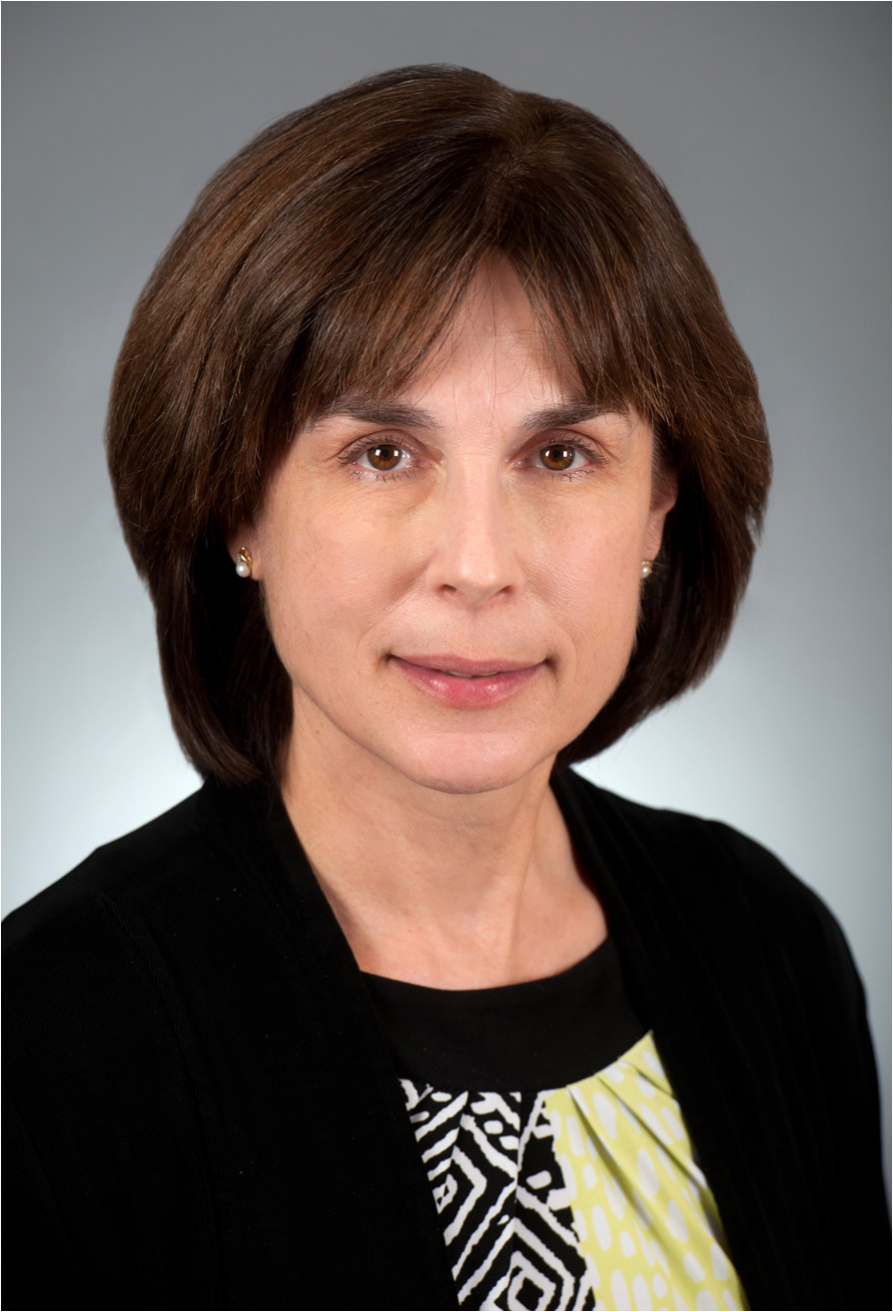
**Adrienne Randolph is a Senior Associate in Critical Care at Boston Children’s Hospital and a Professor of Anesthesia at Harvard Medical School.** She founded the Pediatric Acute Lung Injury and Sepsis Investigator’s (PALISI) Network, a voluntary network of over 80 large pediatric centers across the US and Canada. This network has performed numerous observational studies and clinical trials in children with severe infections. She currently directs Pediatric Intensive Care Influenza and Emerging Severe Pathogens (PICLFU-ESP), a network of investigators with studies funded by the NIH and CDC.

**Figure 6:**
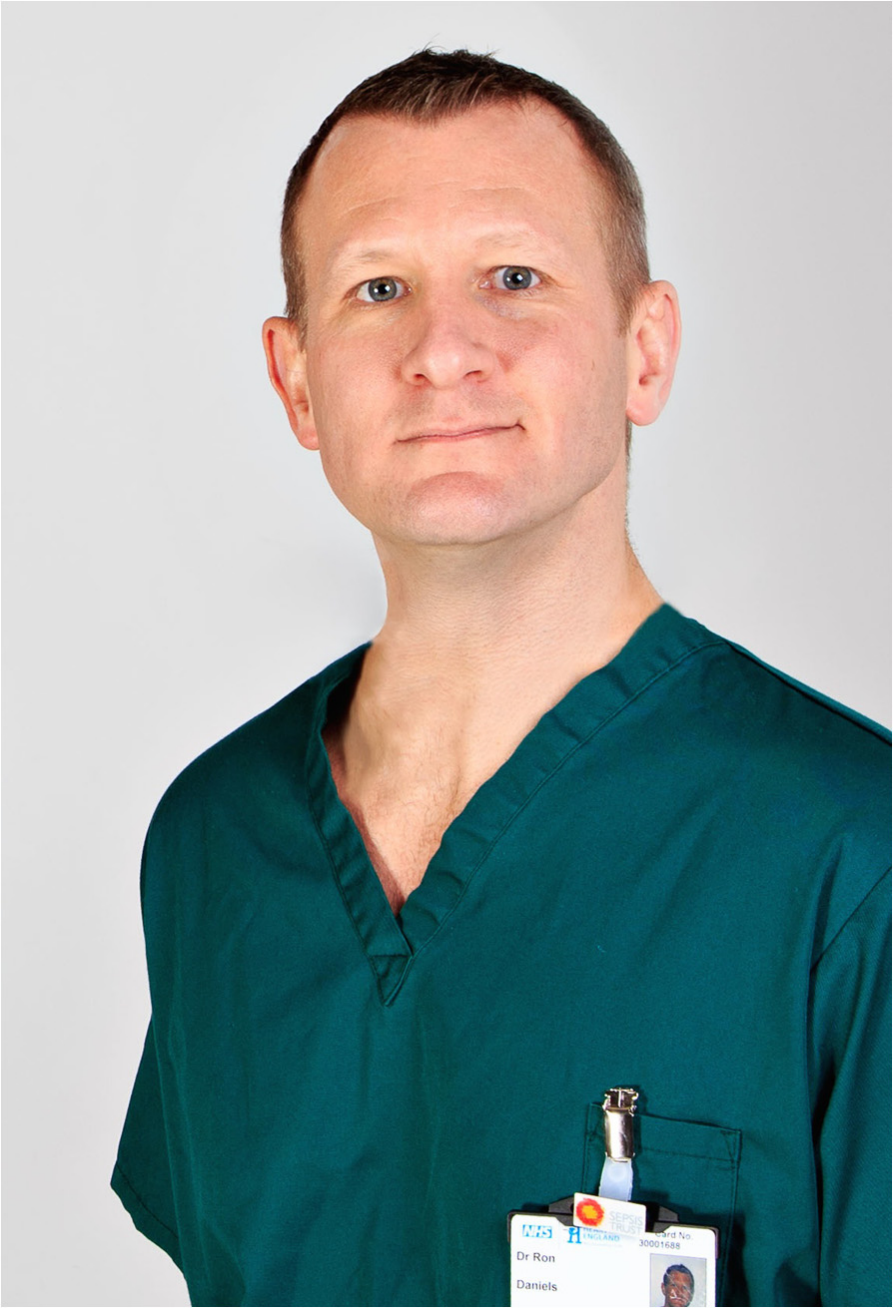
**Ron Daniels is a Consultant in Critical Care based in the United Kingdom (Birmingham).** He is CEO of the UK Sepsis Trust and Global Sepsis Alliance, and Clinical Adviser on Sepsis to NHS England.

**Figure 7:**
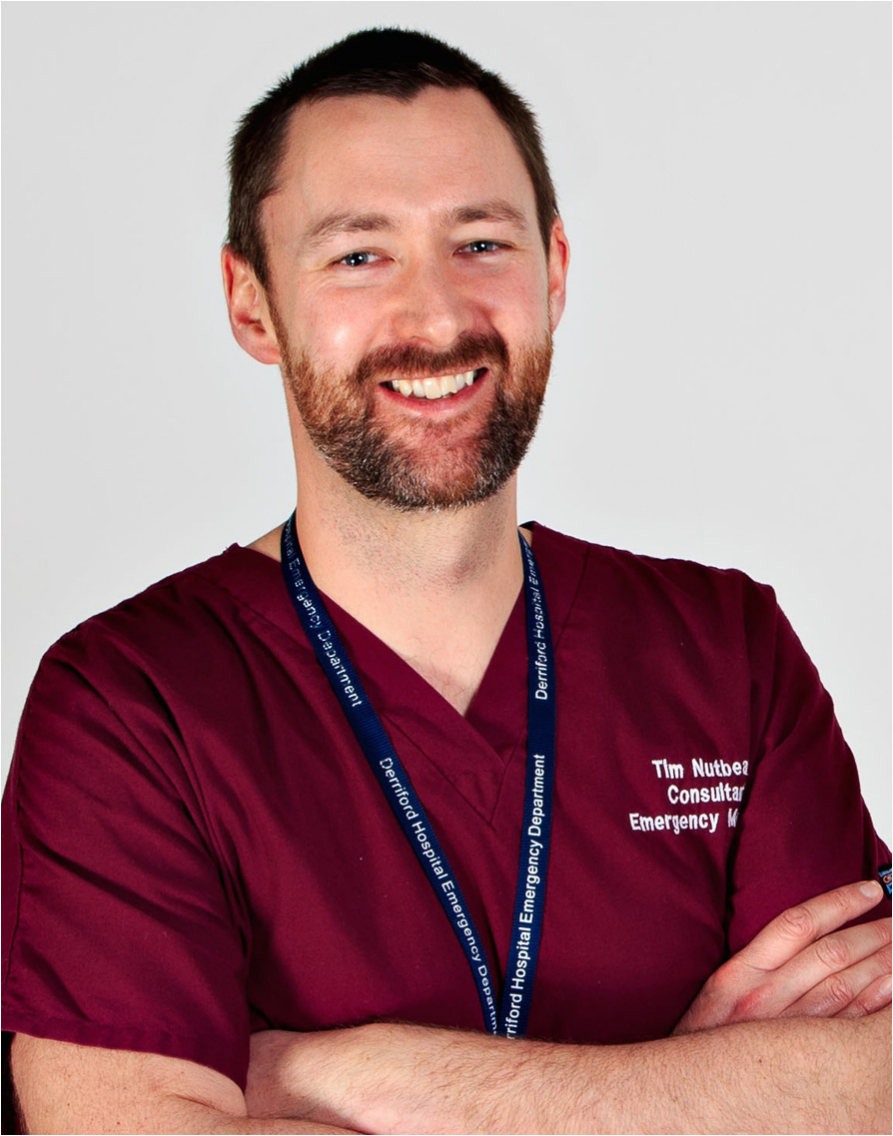
**Tim Nutbeam is a consultant emergency physician and clinical academic based in the United Kingdom (Plymouth).** He is clinical project lead for the UK Sepsis Trust.

Described for centuries, sepsis is a maladaptive inflammatory response to infection, creating profound symptoms and poor outcomes, including high short-term mortality [1,2]. Even into the late 1990s, our experiences, expectations, and insight into the care of the septic patient were dismal and grim. We knew that inflammatory mediators, coagulation, cellular oxygen processing, and both macro- and micro-circulation could be disturbed; the cascading interaction(s) created the dismal outcomes that we dutifully reported and lamented. Half or more of those afflicted died during hospitalization, and we intervened after organ failure was clear using promising biologics that seemed to fix some facets but did not improve mortality or function after recovery.

In 2001, Rivers et al. [3] explored a newer approach, termed Early Goal-Directed Therapy (EGDT). The conceptual model of EGDT was that sepsis and, in some instances, septic shock are under-recognized and hence under-treated. Rather than target mediators and individual organ or cellular events, Rivers et al. [3] sought to limit the global oxygen deficits accompanying sepsis to thwart the cycle of ‘evil humors running amok’ and creating dysfunction. EGDT attempted to achieve this by guiding the first 6 hours of resuscitation with central venous pressure and saturation measures. The structured EGDT approach delivered more fluids (5 L mean) in the first 6 hours, more inotropic support, and more frequent red blood cell transfusion than an unstructured approach. This EGDT use translated into a 16% absolute mortality benefit compared to controls, the most stark noted in sepsis care. Based on this, many called for central catheter-driven EGDT care for all with septic shock [4].

Follow-up work replicated the general observation that earlier recognition, coupled with resuscitative care, improved outcomes [5]. Often, the sites involved had little pre-existing focus on sepsis recognition and care; even when the full EGDT protocol was not implemented – a common event – outcomes improved, suggesting that one path to improved care was possible.

At the same time, others noted a parallel opportunity with another sepsis care target – source control with antibiotics. Delays in delivering appropriate antibiotics led to higher mortality, akin to delayed resuscitation [6]. Coupling these therapeutic insights with the observation that much of the hospitalized sepsis population receives initial care in the emergency department reinforced the importance of early care [7].

Recent research helped clarify the key messages of what early care should be. Jones et al. [8] demonstrated that early resuscitation in the first 6 hours after an aggressive search for sepsis/septic shock using bedside information and serial lactate measurements (to aid resuscitation assessment) was non-inferior to the more regimented and resource-intensive EGDT approach suggested by Rivers et al. [3], delivering hospital mortality rates of 17 to 23%. Our ProCESS trial examined 1,351 patients in 30 US sites, assessing three approaches to resuscitation after early recognition and antibiotic therapy: EGDT with the mandated central oximetric catheter guiding care; a simpler but team-based and clinically-driven protocol; or bedside care tailored by the treating physician (usual care) [9]. The groups received a mean of 4.3 to 5.3 L of fluids through hour 6 of resuscitation and varying amounts of pressor, blood, and inotrope use. Despite the aggressive but varying approaches between those treated in each arm, we observed that how fluids and vasoactive agents were provided did not create a superior outcome as long as each was done early, aggressively, and in the backdrop of early antibiotic use*.* The 60-d hospital mortality amongst the three arms ranged from 18 to 21%, and the secondary outcomes, including organ support and longer term recovery, also did not differ. The ARISE trial, based in Australia/New Zealand, also studied EGDT compared to usual, unstructured care in 1,600 patients with early recognition and infection source control [10]. Like ProCESS, the two different methods of resuscitation in ARISE did not identify one as better and the overall outcomes were improved compared to a decade prior. A separate national surveillance study from Australia/New Zealand confirmed that these lower hospital mortality rates are not simply research or coding aberrations and are due, in part, to earlier care efforts [11].

### Where are we at?

Despite advances, sepsis remains one of the most deadly emergency department arrival or hospital-acquired conditions. Initial sepsis care remains uneven and often sluggish [12]. Long term outcomes show an accrual of death at 1 year after a bout of septic shock exceeding 50% [13] and frequent functional impairment in survivors [14]. While awaiting newer tools to aid detection or treat those who do not respond, we need to focus on wider use of “egdt” – *e*arly and simple detection coupled with the *g*oals of fluids that are adequate and *d*irected by bedside reassessment and appropriate antibiotic *t*herapy – rather than any one singular prescriptive form of resuscitation. Early care matters – not so much a mandated catheter or prescriptive approach – and returning to the days of delayed action is not an option.

### Competing interests

No current industry support in this area. DMY and DTH are funded by NHLBI for the Prevention and Early Treatment of Acute Lung Injury Network (PETAL) and by NIGMS for the Procalcitonin Antibiotic Consensus Trial (ProACT).

## How can we improve the recognition of sepsis?

Anthony Delaney (Figure 3)

Sepsis remains a leading cause of mortality worldwide. While there has been a measureable reduction in mortality over recent years [11], the underlying reasons for this decline in mortality are not entirely clear. More than 100 trials of specific treatments designed to improve mortality in sepsis have failed to demonstrate a mortality benefit [15]. It is possible that further research will lead to a discovery of a novel agent capable of altering the complex interaction between the specific pathogenic mechanisms of various infective agents, the generic host inflammatory reaction to infection, and the pathophysiological response mediated by each individuals’ co-morbidities, in a positive fashion, although such a therapy would appear remote from clinical practice at this point in time. As described in the previous section, the EGDT resuscitation strategy [3] changed the landscape in the management of the early stages of patients with severe sepsis and septic shock. Although subsequent trials [9,10] have not confirmed the benefits of this specific resuscitation algorithm, it is considered that early recognition of sepsis and early application of the fundamental principles of the treatment of sepsis, namely resuscitation, antibiotics, and source control, may have contributed to the gradual decline in mortality over recent times. One of the major challenges facing clinicians is to identify and recognize patients with sepsis and impending organ dysfunction, in the pre-hospital setting, in the emergency department, and for patients who deteriorate in hospital.

Attention to the pre-hospital phase in patients with sepsis is clearly critical. The initial link in this chain is to increase awareness of the symptoms of sepsis amongst the general public. Organizations such as the Global Sepsis Alliance and initiatives like World Sepsis Day, which work to raise awareness of sepsis, play a crucial role in alerting the general public about the importance of seeking medical attention when they or a loved one displays the symptoms of sepsis. Pre-hospital care also plays an important role in recognizing and providing prompt care for patients with sepsis. Approximately 50% of the patients who present to the emergency department with sepsis will arrive via an Emergency Medical Service (EMS) [16]. This exposure provides an excellent opportunity to improve early recognition of sepsis and to commence an integrated system of care for these complex patients. For example, in a pilot prospective cohort study conducted in the pre-hospital setting, early identification of patients with severe sepsis by EMS providers utilizing a screening tool and a point-of-care venous lactate meter was shown to be feasible [17]. Once identified, there is scope for interventions, such as intravenous cannulation and initiation of intravenous fluids provided pre-hospital, to influence the outcomes of patients with sepsis [18].

An enhanced focus on the identification of sepsis in the emergency department setting, as recommended by the Surviving Sepsis Campaign Guidelines [4], has been shown in numerous studies to be associated with improved outcomes [19]. Identifying patients with sepsis in a busy emergency department may be aided by the use of electronic sepsis alert systems [20], or a screening tool that combines simple clinical characteristics with the use of early lactate measurements [21]. Simple tools have been combined in successful large-scale quality improvement programs, such as the Sepsis Kills campaign, run by the Clinical Excellence Commission in NSW, Australia [22], and the Surviving Sepsis Campaign [23]. It is very likely that the increased focus on sepsis by clinical champions, and the awareness amongst front line staff regarding the possibility of sepsis that accompanies such campaigns, is responsible for much of the improved outcomes demonstrated in these programs.

Identifying patients who deteriorate within the hospital secondary to sepsis presents an additional challenge. These populations often have concurrent medical or surgical conditions that confound the diagnosis, making early recognition difficult. The widespread introduction of rapid response systems has led to the early identification and the initiation of early intervention to patients within the hospital system [24], many of whom will deteriorate secondary to sepsis. One other area that offers ongoing promise with regards to the early identification of patients with sepsis is the use of biomarkers. These may be applicable to patients in the emergency department and within the hospital. Traditional individual markers of sepsis, such as the total white cell count, neutrophil count, and C-reactive protein, lack the specificity to allow them to discriminate between those patients with an inflammatory response to trauma or surgery, for example, and those with a new infection. The best studied of the newer biomarkers of sepsis, procalcitonin, which has been investigated in more than 3,000 patients suspected of sepsis [25], does not have sufficient sensitivity to identify all cases of sepsis reliably, nor the specificity to rule out sepsis when negative. Other tests, such as the soluble triggering receptor expressed on myeloid cells-1 (sTREM-1), offer some promise, but still have inadequate sensitivity and specificity to be used as a single biomarker for sepsis. Panels of biomarkers, including sTREM-1, procalcitonin, CD 64 expression on neutrophils, and C-reactive protein, as well as other novel biomarkers, offers some promise as diagnostic markers of sepsis yet to be confirmed in large scale clinical trials [26]. Table 1 summarizes the sensitivity and specificity of selected biomarkers for sepsis.

**Table 1:** **Sensitivity and specificity of selected biomarkers for the diagnosis of sepsis**

	**Sensitivity**	**Specificity**
	**(95% confidence interval)**	**(95% confidence interval)**
C-reactive protein [58]	0.75	0.67
	(0.62–0.84)	(0.56–0.77)
Procalcitonin [25]	0.77	0.79
	(0.72–0.81)	(0.74–0.84)
STREM-1 [59]	0.79	0.8
	(0.65–0.89)	(0.69–0.88)

There are still challenges to be overcome with regards to improving the early recognition of sepsis. The current definition of sepsis [4] remains overly complex and difficult to use in a clinical setting. A new definition, incorporating simple clinical measures, may greatly enhance the evolving clinical science as well as the clinical practice of diagnosing early sepsis. Identifying the best sepsis screening tools, and refining a biomarker or set of biomarkers to definitively identify patients who have sepsis, is a major challenge. These challenges need to be addressed so that, in populations with altered physiology, such as women who are pregnant or in the early post-partum, period, or those in whom clinical features may be non-specific, such as infants and children, the diagnosis of sepsis can be made and the important early interventions can be commenced in an expedient fashion.

### Competing interests

The author was an investigator for the Australasian Resuscitation in Sepsis Evaluation study, a multi-center randomised trial of Early Goal-Directed Therapy, funded by the Australian National Health and Medical Research Council.

## Sepsis in pregnancy and the postpartum period

Marian Knight (Figure 4)

Sepsis is an important cause of both morbidity and mortality in pregnancy and the postpartum period; approximately one in every thousand women giving birth will develop sepsis, with half of these women progressing to severe sepsis and 3 to 4% developing septic shock [27]. Maternal sepsis is a challenge in both low- and high-resource settings; one-quarter of women who die within the 6 weeks after pregnancy in the UK die from sepsis [28]. Globally, an estimated 11% of maternal deaths are caused by sepsis, the vast majority occurring in developing regions [29].

There are several well-recognized risk factors for maternal sepsis, including the presence of pre-existing medical conditions such as anemia, febrile illness in the 2 weeks prior to diagnosis of sepsis, and, most notably, mode of delivery [30]. Cesarean delivery and operative vaginal delivery are both associated with severe maternal sepsis [31]. The predominant causes of maternal sepsis vary according to the timing of infection; antenatally, infections of the urinary tract make up about one-third of all cases of maternal sepsis, whereas postnatally, one-third of sepsis is due to genital tract infections [31]. Overall, infections due to *E. coli* are most numerous, but infections with Group A Streptococcus are significantly associated with greater severity of sepsis [31]. There is good evidence that pregnant women are at higher risk of complications of certain specific infections, for example, influenza, varicella zoster, and listeria.

Pregnant and postpartum women are, in general, young and fit and compensate well, even in the presence of severe infection [32]. Infection may thus be well established before a diagnosis of sepsis is made. Early consideration of the diagnosis and, hence, early recognition is therefore perhaps even more critical in the obstetric population. This is dependent on both the measurement of vital signs in any unwell pregnant or postpartum woman, as well as acting on the findings [28]. Although pregnancy and childbirth is a normal physiological process, normality cannot be assumed in the presence of abnormal signs or symptoms. It is important to note that symptoms, such as severe abdominal pain, breathlessness, and diarrhea, may be associated with postpartum sepsis. Additionally, maternal sepsis should always be considered as part of the differential diagnosis in a postpartum woman presenting with shock. Although hemorrhage is the commonest cause of shock in a postpartum woman, possible sepsis must be investigated, particularly if blood loss is only moderate and treatment for hemorrhage appears ineffective.

In relation to maternal shock, it is important to be aware of the significance of Group A Streptococcal infection. Although cesarean delivery and operative vaginal delivery are both risk factors for maternal sepsis, Group A Streptococcal infections are proportionately more frequently seen in women who have had an unassisted vaginal delivery. Group A Streptococcal infection is a known risk factor for development of septic shock, and is associated with rapid sepsis progression [31]. In a UK national study of severe maternal sepsis, for 75% of women with a Group A Streptococcal infection there was less than 9 hours between the first signs of systemic inflammatory response syndrome and the diagnosis of severe sepsis [31]. In 50% of women this was less than 2 hours.

As with sepsis in general populations, along with early recognition and diagnosis, the key action for management is to institute a sepsis care bundle, including administration of timely antibiotics (within 1 hour of suspected sepsis), adequate fluid resuscitation, and the measurement of serum lactate [28]. There is no specific sepsis care bundle suggested for use in pregnancy or the postpartum period; several have been recommended, including the Surviving Sepsis Campaign resuscitation care bundles [4], the Institute for Healthcare Improvement severe sepsis bundles [33], and the ‘Sepsis Six Care Bundle’ from the UK Sepsis Trust [34]. The choice of antibiotic is dependent on the likely source of infection, taking into account known hospital and individual factors such as prevalence of antibiotic resistant organisms and mode of delivery. Adjustment may be made later based on the woman’s response and subsequent culture results.

Source control is essential in the timely management of maternal sepsis; bearing in mind that this may require cesarean delivery, hysterotomy, or hysterectomy in women with genital tract sepsis. The UK and Ireland Confidential Enquiry into Maternal Deaths identified a number of women who subsequently died from maternal sepsis following delayed delivery [28]. In several women, delivery was delayed because the fetus had already died and there was a perceived need to ensure the woman delivered vaginally. Failure to deliver the fetus and placenta early in this situation will lead to a persisting source of infection and progression of the woman’s sepsis despite adequate resuscitation and antibiotic treatment.

Some evidence suggests that rates of maternal sepsis may be increasing [27], and with more than an estimated 30,000 maternal deaths from sepsis annually [29], ongoing awareness of the diagnosis, timely recognition, and management is vital.

### Competing interests

The author declares that she has no competing interests.

## Recognizing and managing pediatric sepsis

Adrienne G. Randolph (Figure 5)

Overwhelming infection is the major cause of death in children worldwide [35]. Neonates and young infants are at highest risk because their immature immune systems are less able to ward off severe pathogens [35,36]. Sepsis – when a patient has a systemic inflammatory response to a suspected or confirmed infection [37] – is a useful construct in that it should bring the clinician to the bedside to examine the child and determine the proper treatment course. Children who develop signs of severe sepsis with organ dysfunction, and especially those who develop septic shock, are at highest risk of life-threatening and fatal complications. In recent years, the approach to treating septic shock in the pediatric patient has focused on early recognition of severe sepsis. This is followed by rapid treatment with antibiotics and aggressive treatment of shock with fluid boluses and, if refractory, with vasoactive agents. These interventions, extrapolated from the care of septic adults, have been widely disseminated as clinical guidelines [4]. Implementation of these interventions as care bundles with audit and feedback can optimize clinician compliance [38]. Although no large multi-center randomized trials have been performed, there is some evidence that implementation of early sepsis recognition, prompt delivery of antibiotics, and intravenous fluids have improved clinical outcomes [4]. There is very strong evidence that focused attention on constantly adhering to the use of central-line insertion and maintenance bundles can prevent nosocomial sepsis from central line-associated bloodstream infections [39].

Early sepsis recognition and aggressive treatment are necessary but insufficient. Treatment of ‘sepsis’ should rapidly evolve into a specific diagnosis with targeted antimicrobial therapy against the bacterial, viral, fungal or protozoal invading pathogen. The most common infection in children is pneumonia, the majority caused by viral pathogens [40]. Therefore, despite their widespread use, antibiotics are ineffective for most children with a severe infection. With the exception of influenza, antivirals are also usually not indicated in immune competent hosts. The rapid implementation of strategies aimed at decreasing use of antibiotics is also not without risk. Children with life-threatening viral infections are frequently co-infected with highly pathogenic bacteria. In recent years, methicillin-resistant *Staphylococcal aureus* or MRSA, which used to be relatively rare in children, has taken a rising toll; influenza-MRSA is an especially fatal combination [41].

Although pediatric sepsis is common, no large-scale epidemiologic study has been performed to refine the widely used pediatric sepsis diagnostic criteria [37]. These criteria were originally developed for identifying children eligible to participate in a drug trial and not for clinical management. Tachypnea, tachycardia, fever, and alterations in leucocyte count are sensitive indicators, but they encompass many non-infectious acute inflammatory disorders. Delayed capillary refill, lethargy, and other signs of severe sepsis are also highly relevant but non-specific. Which children should be given fluid, what type of fluid, and how much, are all areas that merit urgent study. A recent study of aggressive fluid resuscitation in African children with compensated shock revealed worse outcomes in those who received fluid boluses with albumin or saline [42], a surprising finding that was difficult to understand. Whether aggressive fluid resuscitation in children with bronchiolitis or pneumonia that present with poor capillary refill and other signs of compensated shock improves or worsens their outcome is yet to be evaluated.

Rigorous implementation of the Surviving Sepsis guidelines will save many lives, but the broad definition of sepsis makes it likely that the number of children exposed to potentially unnecessary treatments will also increase. An informed and targeted approach to the diagnosis and management of the infections subsumed within the term ‘sepsis’ is required to optimize clinical outcomes. This requires cost-effective implementation of newly available validated rapid diagnostic tests. Therapy could be streamlined, exposing children only to those antimicrobial agents effective against the identified pathogen. Immunodiagnostics could also help guide immunomodulatory therapies [43,44]. Optimizing outcomes in pediatric sepsis requires rigorous research to identify those children that could benefit from rapid diagnostic testing and targeted antimicrobial treatment and those for whom this is unnecessary.

### Competing interests

Dr. Randolph is on the Scientific Advisory Board for Ferring, Inc., Asahi Kasei Pharma, Inc. and Genentech, Inc. advising on the design of pediatric trials for sepsis agents.

## The importance of identifying sepsis in the pre-hospital setting

Ron Daniels (Figure 6) and Tim Nutbeam (Figure 7)

Patients with sepsis are commonly transported to emergency departments by EMS [18,45,46]. These patients tend to have more severe sepsis and have a higher mortality than those who present by other methods [47]. As discussed in the previous sections, prompt identification and early treatment reduces the mortality and morbidity associated with sepsis. It has been demonstrated in the in-hospital setting that the earlier this identification and treatment delivery can occur the better a patient’s outcomes will be [48]. With an average pre-hospital care interval in excess of 45 minutes for patients with sepsis, early intervention should occur in the pre-hospital setting [16].

Sepsis has not been subject to the public health awareness campaigns that stroke and acute coronary syndromes have benefited from; as such, public awareness is low. A recent poll conducted by the UK Sepsis Trust identified that only 16% of the public were aware that sepsis was a time-critical condition [49]. These findings mirror similar surveys conducted in Germany and the USA.

Awareness amongst EMS professionals is also low, with less than 10% of pre-hospital providers having knowledge and skills sufficient to pass sepsis-focused scenarios in a recent survey [50]. This may, in part, be due to the lack of pre-hospital sepsis dispatch and clinical care protocols or guidelines in many regions [51,52]. Skills availability is also an issue; only about 50% of patients transported by an EMS system with severe sepsis will have a paramedic present [16]. The lack of a trained paramedic will impact at all stages of patient care, including awareness of sepsis (and its mimics), ability to screen a patient for the presence of sepsis, and ability to intervene (e.g., provide intravenous fluids).

There are a number of ways in which sepsis recognition can be improved in the pre-hospital setting. EMS practitioners should use a screening tool [51] to identify patients with sepsis. This should trigger the rapid delivery of effective protocolized care [53,54]. Lactate may be used to risk stratify patients effectively in the pre-hospital setting [17]. Unfortunately, few EMS systems have established sepsis care bundles, with the delivery of antibiotics outside of treatment protocols for suspected meningococcal sepsis being rare [51]. Even when protocols are available, EMS crews only successfully identify patients 21% of the time [47]. Delivery of intravenous fluid administration is achieved in less than half of cases (and intravenous cannulation in only one-third) with those who do receive fluid not demonstrating a decreased mortality [16,55].

Delivery to an emergency department by an EMS system is associated with improved in-hospital processes, demonstrated by a decreased time to antibiotics and an increased volume of intravenous fluid therapy [56], with these processes taking even less time when sepsis was identified in the pre-hospital environment [47,55]. Despite emergency department and in-hospital studies repeatedly demonstrating a decreased mortality with early care, early EMS identification and treatment have not consistently been associated with improved outcomes in this patient group [16,51,57] – placing a pre-hospital IV catheter and delivering fluids does, however, appear to be associated with a better outcome [18].

Improvement strategies are needed to increase early sepsis awareness in pre-hospital care at each step of the process. National and regional strategies should focus on the steps outlined in Box 1, and further details about clinical tools from the UK Sepsis Trust are given in Table 2.

**Table 2:** **The Sepsis Six and Red Flag Sepsis, clinical tools from the UK Sepsis Trust**

**Sepsis Six**	**Red Flag Sepsis**
1 High-flow oxygen	Systolic BP <90 mmHg or MAP <65 mmHg
2 Blood cultures and consider source control	Lactate >2 mmol/L
3 Intravenous antibiotics	Heart rate >130 per minute
4 Intravenous fluid resuscitation	Respiratory rate >25 per minute
5 Check hemoglobin and serial lactates	Oxygen saturations <91%
6 Hourly urine output measurement	Responds only to voice or pain/unresponsive
	Purpuric rash
An immediate care bundle to be delivered within one hour for patients with sepsis	A risk stratification tool for patients with possible sepsis

### Competing interests

Ron Daniels and Tim Nutbeam both believe sepsis is an under-diagnosed, time critical condition that responds well to early judicious therapy: they have established a charity to improve sepsis care in the UK and further afield (the UK Sepsis Trust) – they have no financial conflicts of interest.

## Box 1: Recommendations for national and regional strategies to improve sepsis awareness in the pre-hospital setting

- Widespread public awareness campaigns with clear triggers for contacting medical or emergency services. Much can be learnt from the successes of campaigns targeting stroke and acute coronary syndromes.
- Pre-hospital practitioner education should include ‘sepsis’ as a significant curriculum element.
- Clear triggers to initiate formal ‘sepsis screening’ by pre-hospital teams and community practitioners: this screening may be triggered by a clinical observation-based ‘Early Warning Score’ or equivalent. This screening should result in a binary, documented decision of the presence or absence of sepsis.
- Patients should be risk stratified using clinical observations (e.g., ‘Red Flag Sepsis’ from the UK Sepsis Trust (Table 2) or alternative markers, e.g., lactate. High-risk patients should be pre-alerted to the receiving emergency department.
- Initial resuscitation should begin within the capabilities of the pre-hospital team – this may be a modified ‘Sepsis Six’ (Table 2) or equivalent.
- The management of pre-hospital sepsis should be subject to the same measurement and targets as those cases identified in the in-hospital environment, e.g., time to antibiotic quality standard.

## Abbreviation

EMS: Emergency medical services
